# Intracranial electroencephalographic connectivity analysis to localize epileptogenic networks: Systematic review and meta‐analysis from ILAE Epilepsy Surgery Networks Task Force

**DOI:** 10.1002/epi.70168

**Published:** 2026-03-02

**Authors:** Nishant Sinha, Daniel J. Zhou, Victoria L. Morgan, Dario J. Englot, Leonardo Bonilha, Milan Brázdil, Xiaosong He, Riki Masumoto, Quy Cao, Terence J. O'Brien, Chengyuan Wu, Erik Kaestner, Carrie McDonald, Ezequiel Gleichgerrcht, Aileen McGonigal, Kathryn A. Davis

**Affiliations:** ^1^ Department of Biostatistics, Epidemiology, and Informatics University of Pennsylvania Philadelphia Pennsylvania USA; ^2^ Department of Neurology University of Pennsylvania Philadelphia Pennsylvania USA; ^3^ Department of Biomedical Engineering Vanderbilt University Medical Center Nashville Tennessee USA; ^4^ Department of Neurological Surgery Vanderbilt University Medical Center Nashville Tennessee USA; ^5^ Department of Neurology University of South Carolina Columbia South Carolina USA; ^6^ Brno Epilepsy Center, First Department of Neurology, St. Anne's University Hospital and School of Medicine Masaryk University Brno Czech Republic; ^7^ Department of Psychology University of Science and Technology of China Hefei China; ^8^ Department of Neurology Kyoto University Kyoto Japan; ^9^ Department of Neuroscience, School of Translational Medicine, Alfred Health and Monash University Melbourne Victoria Australia; ^10^ Department of Neurosurgery Thomas Jefferson University Philadelphia Pennsylvania USA; ^11^ Departments of Radiation Medicine & Applied Sciences and Psychiatry University of California, San Diego San Diego Georgia USA; ^12^ Department of Neurology Emory University Atlanta Georgia USA; ^13^ Faculty of Health, Medicine and Behavioural Sciences University of Queensland Brisbane Queensland Australia

**Keywords:** connectivity, epileptic networks, iEEG, localization, surgery

## Abstract

Intracranial electroencephalographic (iEEG) connectivity analysis is a promising method to localize epileptic networks and guide surgical planning in focal drug‐resistant epilepsy. Despite numerous studies exploring its utility, the added value of iEEG connectivity over standard clinical presurgical evaluation remains unclear. We assess the current evidence on the efficacy of iEEG connectivity analyses to improve seizure outcomes following epilepsy surgery through a systematic review and meta‐analysis. Following PRISMA (Preferred Reporting Items for Systematic Reviews and Meta‐Analyses) reporting guidelines, we searched PubMed and Embase for studies (2006–2024) of adult focal drug‐resistant epilepsy patients who underwent surgical resection or ablation, reported outcomes at least 1 year postsurgery, and used iEEG connectivity analysis to localize networks. Reviews, nonhuman studies, and studies lacking iEEG connectivity analysis or network localization were excluded. We derived classification metrics (true/false positives/negatives) based on concordance between iEEG findings, clinical localization, and outcome. Subgroup meta‐analyses and meta‐regressions determined differences by seizure type, lesion status, and analysis approach. Of 2881 studies screened, 25 met criteria (*n* = 909). The pooled odds ratio comparing seizure outcome prediction using iEEG connectivity versus standard clinical evaluation was 1.36 (95% confidence interval = 1.10–1.69, *p* = .004), indicating a significant overall benefit. Subgroup analyses found no significant differences by directionality, modeling method (linear/nonlinear), or iEEG epoch (interictal/peri‐ictal). Meta‐regression revealed greater added value of iEEG connectivity in studies with higher proportions of non‐seizure‐free patients following surgery for temporal lobe or lesional epilepsy. However, no individual study achieved statistical significance on its own, reflecting limited power and lack of individual patient‐level data. Power analysis confirmed that detecting a clinically meaningful effect requires substantially larger, potentially multicenter datasets. iEEG connectivity analysis offers modest but consistent increased value over standard clinical methods to predict seizure freedom in adult patients with focal drug‐resistant epilepsy. For clinical translation, we propose recommendations for future studies to address sample size limitations, standardize reporting, and prioritize individual patient‐level data sharing.

**Key points**

- iEEG connectivity provides modest but consistent added value over standard clinical evaluation for epilepsy surgery planning.
- Methodological heterogeneity limits translation, underscoring the need for standardized reporting and patient‐level data sharing.
- Large multicenter datasets are required to validate iEEG connectivity as a clinically actionable tool.

## 1 INTRODUCTION

Epilepsy surgery has the potential to control seizures in a proportion of up to one third of the 70 million people worldwide with epilepsy whose seizures are resistant to antiseizure medications, but only when epileptogenic regions are accurately identified and targeted.^1^, ^2^ Currently, surgical planning relies on a comprehensive, case‐by‐case synthesis of clinical history, seizure semiology, and multimodal diagnostic data from noninvasive and invasive imaging and electrophysiology. Despite these efforts, 40%–60% of patients continue to experience seizures after surgery, underscoring the limitations of current approaches.^3^ These limitations have fueled interest in quantitative methods for mapping epileptic brain networks, with the goal of improving surgical targeting and predicting which patients are most likely to benefit from intervention.^4^

Intracranial electroencephalographic (iEEG) connectivity analysis has emerged as a promising tool to quantitatively localize epileptic networks.^5^ This approach treats each electrode contact as a node and uses statistical measures—such as correlation and information flow, among others—to estimate functional connections between nodes, thereby constructing a functional network model of brain activity from iEEG.^6^, ^7^, ^8^, ^9^ Over the past 2 decades, numerous studies have demonstrated that analyzing these networks, using graph theory or dynamical models, can help identify epileptogenic regions and may improve surgical planning and targeting. However, despite growing scientific interest, clinical implementation of iEEG connectivity analysis remains limited.^10^ One notable initiative is the EPINOV clinical trial in France, which integrates iEEG‐derived connectivity maps with computational modeling to guide surgical decisions.^11^ Nonetheless, to date, no systematic review or meta‐analysis has quantified the added value of iEEG connectivity analysis compared to standard clinical evaluation in predicting postoperative seizure outcomes.

The primary objective of this study was to evaluate the added value of iEEG connectivity analysis over standard clinical approaches in predicting postoperative seizure outcomes in adult patients with focal drug‐resistant epilepsy. To achieve this, we conducted a systematic review and meta‐analysis of studies that used iEEG connectivity methods to localize epileptic networks. Beyond quantifying the predictive performance of iEEG connectivity methods, we also examined sources of variability in patient characteristics, connectivity approaches, and analytic pipelines. Our work identifies methodological gaps and barriers to clinical translation to inform standardized reporting practices and supports future efforts toward multicenter collaboration and prospective clinical trials.

## 2 MATERIALS AND METHODS

**FIGURE 1:**
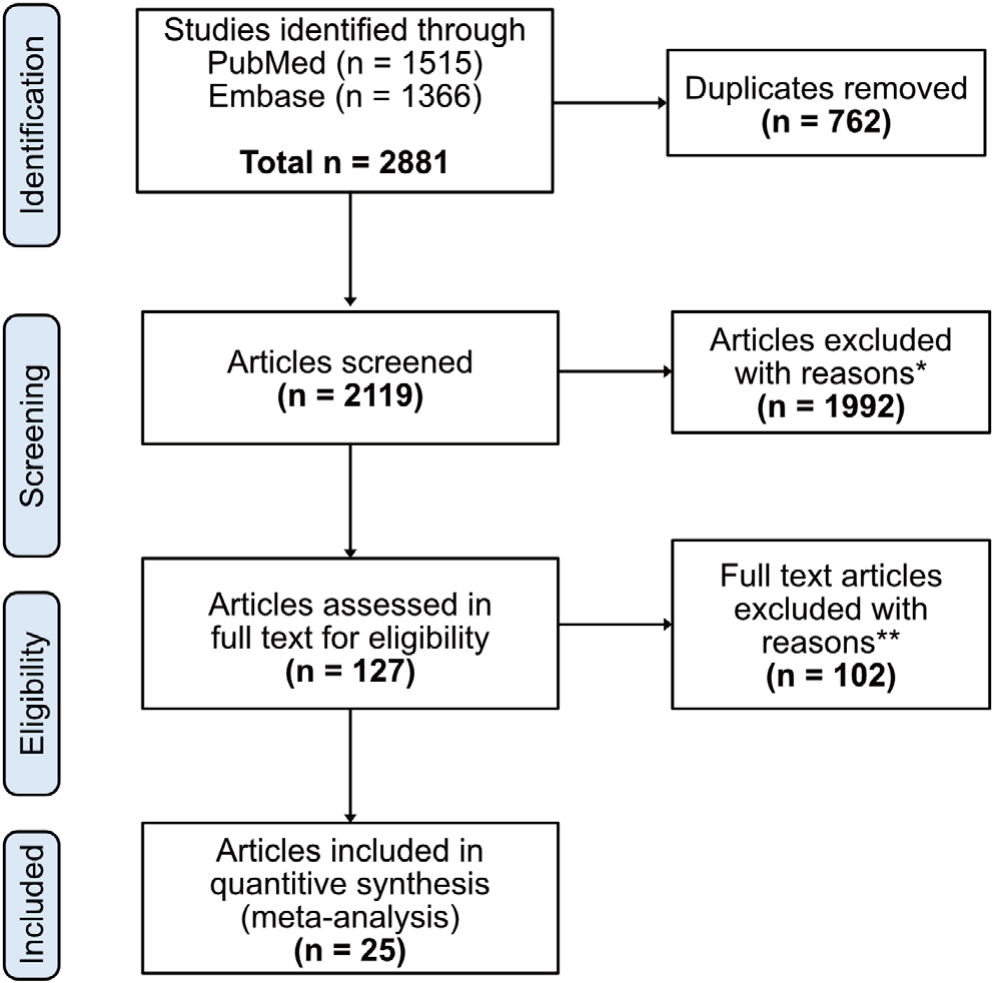
PRISMA (Preferred Reporting Items for Systematic Reviews and Meta‐Analyses) flow diagram of selection process for articles included in the meta‐analysis. *Studies did not meet inclusion and exclusion criteria. **Reasons for exclusion were as follows: outcome not reported on the Engel or International League Against Epilepsy scale (*n* = 20); study did not perform intracranial electroencephalographic connectivity analysis (*n* = 20); not a peer‐reviewed research article (*n* = 16); limited data, reporting only summary statistics without individual‐level data points or contingency tables (*n* = 14); pediatric population (*n* = 11); wrong comparator (*n* = 11); multiple studies on overlapping populations (*n* = 4); five or fewer subjects (*n* = 3); follow‐up <1 year after surgery (*n* = 2); not in English (*n* = 1).

This review was registered prospectively with the PROSPERO (Prospective Register of Systematic Reviews) registry of systematic reviews (CRD42023446587). The Preferred Reporting Items for Systematic Reviews and Meta‐Analyses (PRISMA) standards were followed (Figure 1).

### 2.1 Information sources and search strategy

We systematically searched PubMed and Embase databases using a strategy developed in collaboration with a medical librarian at the University of Pennsylvania. The search included a combination of controlled vocabulary terms (MeSH and Emtree) and free‐text keywords targeting epilepsy, surgery, and iEEG connectivity (see Text S1 for full strategy). The initial search was conducted on August 29, 2023 and updated on December 15, 2024. We included studies published from January 2006 onward, corresponding with the formal introduction of network neuroscience frameworks.^12^ We did not include gray literature (e.g., conference proceedings, preprints).

### 2.2 Eligibility criteria

We included studies involving adult patients with focal drug‐resistant epilepsy who underwent surgical resection or ablation of clinically determined seizure generators. Eligible studies were required to incorporate iEEG connectivity analysis to localize epileptic networks and to report postoperative seizure outcomes using the Engel or International League Against Epilepsy (ILAE) classification systems with a minimum follow‐up of 1 year.^13^ We defined connectivity analysis as a method in which nodes and edges were formulated from iEEG data and quantified with network graph models. The comparator was the standard clinical evaluation operationalized as the surgical targets (i.e., areas removed using resection or ablation therapy), which were defined by interdisciplinary presurgical epilepsy conferences. In cases of multiple publications from the same center with overlapping patient populations, we retained the study that incorporated a distinct iEEG connectivity approach or differed meaningfully in the type of data analyzed (e.g., peri‐ictal vs. interictal segments). We also excluded studies in which postoperative outcomes were not reported using the Engel or ILAE classification systems; studies that did not perform iEEG connectivity analysis; studies that were not peer‐reviewed research articles; studies with limited data, reporting only summary statistics without individual‐level data points or contingency tables; studies focused on pediatric populations; studies with an inappropriate comparator (i.e., focus was not on surgical resection or ablation to control seizures or localization of seizure onset); studies based on overlapping patient populations; studies including five or fewer subjects; studies with follow‐up shorter than 1 year after surgery; and studies not published in English.

### 2.3 Study selection, data collection, and extraction

The title and abstract of each study were independently screened by two reviewers randomly selected from the author list, with conflicts adjudicated by a third reviewer (N.S. or E.G.). Studies found to be possibly relevant by either reviewer were retained for a second screen. Full texts of the retained studies were then independently screened by two reviewers (N.S., D.J.Z.) and conflicts were resolved by discussion or with the help of a third reviewer (K.A.D.).

Data were extracted independently by two reviewers (N.S., D.J.Z.) using a standardized protocol with a data extraction form, with discrepancies resolved by consensus or a third reviewer (K.A.D.). Eligible studies were required to report either individual patient‐level data or sufficient information to reconstruct a contingency table from clearly displayed plots or reported sensitivity, specificity, or accuracy values.^14^ Extracted variables included study details, cohort characteristics (sample size), epilepsy type (e.g., temporal vs. extratemporal), lesion status, iEEG modality (e.g., stereo‐EEG [SEEG], electrocorticography [ECoG]), type and time segments of iEEG connectivity analysis (e.g., directed vs. undirected, interictal vs. peri‐ictal data), surgical intervention, and seizure outcomes based on Engel or ILAE classification. Definitions of good and poor outcomes varied across studies; some studies considered Engel I (ILAE 1–2) to be a good outcome, whereas others considered Engel IA–IB (ILAE 1) as a good outcome. To pool individual patient data across studies, we defined Engel I (ILAE 1–2) as a good outcome and others as poor outcomes.

The primary objective of this meta‐analysis was to evaluate the added value of iEEG connectivity methods over standard clinical approaches in predicting seizure outcomes following surgery for focal drug‐resistant epilepsy. To allow for comparison across studies, we extracted or derived classification outcomes at the optimal threshold separating good and poor surgical outcomes, specifically, the number of true positives (TP), false positives (FP), false negatives (FN), and true negatives (TN) based on the concordance between iEEG connectivity findings and clinical localization.

TN were defined as cases in which the epileptogenic areas identified by iEEG connectivity analysis concorded with clinical localization and the patient achieved a good seizure outcome. These cases were classified as supportive of iEEG connectivity methods. In contrast, FN referred to patients who achieved good outcomes, but whose iEEG connectivity‐derived targets did not concord with clinical localization, indicating that the method did not contribute meaningfully to localization. FP referred to patients who had poor surgical outcomes despite concordance between iEEG connectivity findings and clinical localization. Both FP and FN outcomes were considered unfavorable for iEEG connectivity, as they failed to demonstrate added value beyond standard clinical methods.

TN cases were those in which iEEG connectivity findings did not concord with clinical localization and the patient had a poor outcome. However, these cases were excluded from performance assessment, as retrospective analysis cannot determine whether targeting iEEG connectivity suggested regions would have improved outcomes. Given the absence of a defined ground truth for alternative surgical targets, TN cases were treated as indeterminate and not included in final calculations, consistent with prior methodological frameworks.^15^ Figure S1 and Supplementary Glossary illustrate these scenarios with example cases and additional explanation.

For each study, we calculated two performance metrics. The success rate of clinical methods was defined as the ratio of good outcome cases to the total number of patients treated. The success rate of the iEEG connectivity method was calculated as the proportion of TP relative to the sum of TP, FP, and FN (i.e., TP / [TP + FP + FN]). We pooled these metrics across studies and computed the odds ratio (OR) to quantify the added value of iEEG connectivity methods over standard clinical approaches in localizing epileptic networks to determine postoperative seizure freedom.

Different studies used different connectivity analysis methods, which may contribute to variability in performance.^8^ Rather than benchmarking specific connectivity analysis techniques, this meta‐analysis focuses on their shared clinical goal: quantitative localization of epileptic networks to improve surgical planning. By summarizing classification outcomes and computing ORs, we quantitatively assessed the added value of iEEG connectivity across heterogeneous methodologies. This approach allows for meaningful comparison across studies while treating study‐specific characteristics, including the connectivity method used, as random effects in the meta‐analysis.

Importantly, this analysis does not compare iEEG connectivity against clinical evaluation as independent alternatives. iEEG connectivity analyses are inherently dependent on clinical decisions, particularly in electrode placement, which is guided by standard clinical evaluations. Therefore, the goal of this meta‐analysis is to assess the incremental value of incorporating connectivity analysis to complement, not replace, clinical decision‐making. We do not imply that iEEG connectivity methods are superior to standard clinical approaches but rather explore how they may contribute additional insights within a multidisciplinary evaluation framework.

### 2.4 Risk of bias assessment

The risk of bias for each study was evaluated independently by two authors (N.S. and D.J.Z.) using the Joanna Briggs Institute Critical Appraisal Scale for case series, case–control, and cohort studies, depending on study design (Table S1).^16^ Discrepancies were again resolved by discussion with unanimous agreement.

### 2.5 Meta‐analysis

We conducted the meta‐analysis using the open access *xmeta* package in the R statistical environment, developed by the Department of Biostatistics, Epidemiology, and Informatics at the University of Pennsylvania. This framework integrates functionalities from established R packages (e.g., *meta*, *metafor*) and Stata‐like routines to offer comprehensive options for modeling and visualization. Effect sizes were modeled as log ORs with corresponding sampling variances, using outcome data to compare seizure freedom rates between iEEG connectivity and standard clinical methods. We applied a random effects model, assuming heterogeneity across studies, and estimated between‐study variance using restricted maximum likelihood. Summary estimates were presented as pooled ORs with 95% confidence intervals (CIs). To examine sources of between‐study variation, we performed random effects meta‐regression using the *metafor* package. Moderators included the proportion of non‐seizure‐free patients stratified by epilepsy type (temporal vs. extratemporal) and lesion status (lesional vs. nonlesional). To account for small‐sample bias and potential heteroscedasticity, we used robust variance estimation via cluster‐robust sandwich estimators, treating each study as a cluster. We conducted predefined subgroup analyses based on EEG epoch type (interictal vs. peri‐ictal), connectivity modeling approach (linear vs. nonlinear), and directionality (directed vs. undirected). Between‐subgroup differences were assessed using *χ* ^2^ tests. Finally, to assess publication bias, we visually inspected funnel plots and performed Egger regression test. To evaluate the potential impact of small‐study effects, we applied the Trim and Fill method, estimating the number of potentially missing studies and providing adjusted pooled estimates. All analyses were performed in R (version 4.4.1), and statistical significance was defined as *p* < .05 (two‐tailed). Bubble plots and forest plots were generated using the *xmeta*, *ggplot2*, and *metafor* packages to visualize effect sizes, heterogeneity, and meta‐regression results.

## 3 RESULTS

From 2881 records, 25 studies met inclusion criteria, contributing a total of 909 adult patients with focal drug‐resistant epilepsy (PRISMA, Figure 1). Across these studies, temporal lobe epilepsy (TLE) accounted for approximately 69% (*n* = 628) and extratemporal or mixed localization for 31% (*n* = 281). Lesional cases comprised 61% (*n* = 554) and nonlesional cases 39% (*n* = 355). Most studies (16/25, 64%) used SEEG, whereas nine (36%) used ECoG. Connectivity analyses included directed (10/25) and undirected (15/25) approaches, most derived from interictal data (18/25). All studies reported postoperative outcomes using Engel or ILAE classifications with ≥12‐month follow‐up. Aggregate study characteristics are summarized in Table 1.

**TABLE 1:** Patient demographics and connectivity methods from articles included in meta‐analysis.

Study	Patients, *n*	Clinical characteristics				iEEG connectivity method	
		Seizure‐free, *n*	Localization: TLE (ETLE), *n*	MRI lesional (nonlesional), *n*	Implant type	Epoch	Connectivity type
Antony 2013^17^	23	10	10 (13)	15 (8)	SEEG	Interictal	Undirected Cross‐correlation
Epstein 2014^18^, ^a^	11	6	11 (0)	‐	ECoG	Peri‐ictal	Directed Granger Causality
Sinha 2017^19^	16	8	10 (6)	‐	ECoG	Interictal	Undirected Cross‐correlation
Wang 2017^20^	43	29	43 (0)	29 (14)	SEEG	Interictal	Directed Phase transfer entropy
Grobelny 2018^21^	36	22	‐	‐	ECoG	Interictal	Directed Autoregressive model
Lopes 2018^22^	16	6	12 (4)	9 (7)	ECoG	Peri‐ictal	Undirected Cross‐correlation and dynamical network model
Lagarde 2018^23^	59	34	‐	51 (8)	SEEG	Interictal	Directed *h* ^*2*^ regression
Kini 2019^24^	28	17	22 (6)	11 (17)	ECoG	Peri‐ictal	Undirected Cross‐correlation
Cimbalnik 2019^25^	28	18	‐	‐	SEEG	Interictal	Undirected Multiple metrics combined
Zweiphenning 2019^26^	18	12	6 (12)	18 (0)	ECoG	Interictal	Directed Autoregressive
Shah 2019^27^	27	16	18 (9)	‐	ECoG	Interictal	Undirected Cross‐correlation
Klimes 2019^28^	30	13	13 (17)	13 (17)	SEEG	Interictal	Undirected Multiple metrics combined
Wang 2020^29^	55	20	35 (20)	‐	SEEG and ECoG	Interictal	Undirected Cross‐correlation
Narasimhan 2020^30^	18	9	‐	‐	SEEG	Interictal	Directed Multiple metrics combined
Gunnarsdottir 2022^31^	65	28	28 (37)	19 (46)	SEEG	Interictal	Directed Autoregressive model
Bernabei 2022^32^	60	38	42 (18)	29 (31)	SEEG and ECoG	Interictal	Undirected Coherence standardized with normative atlas
Jiang 2022^33^	27	19	23 (4)	12 (15)	SEEG	Interictal	Directed Information flow
Hu 2023^34^	53	39	21 (32)	39 (14)	SEEG	Peri‐ictal	Directed *h* ^2^ regression
Wang 2023^35^	25	12	18 (7)	16 (9)	SEEG	Interictal	Undirected Cross‐correlation and dynamical network model
Shen 2023^36^, ^b^	47	29	24 (17), 18 unspecified	‐	SEEG and ECoG	Interictal	Undirected Skewness metric
Sinha 2023^37^	39	15	22 (17)	23 (16)	SEEG and ECoG	Interictal	Undirected Cross‐correlation in areas structurally connected
Makhalova 2023^38^	53	31	26 (27)	34 (19)	SEEG	Peri‐ictal	Directed *r* ^2^ regression
Wang 2024^39^	17	10	‐	7 (10)	ECoG	Peri‐ictal	Directed Convergent cross‐mapping
Gong 2024^40^	69	45	54 (15)	33 (38)	SEEG and ECoG	Interictal	Undirected Coherence
Chen 2025^41^	46	29	‐	21 (25)	SEEG and ECoG	Peri‐ictal	Directed Phase transfer entropy

Abbreviations: ECoG, electrocorticography; ETLE, extra‐temporal lobe epilepsy; iEEG, intracranial electroencephalographic; MRI, magnetic resonance imaging, SEEG, stereoelectroencephalography; TLE, temporal lobe epilepsy.

^a^ Included two prospective cases.

^b^ Excluding cases where resection or ablation was not performed.

### 3.1 Meta‐analysis summary of iEEG connectivity versus clinical evaluation

**FIGURE 2:**
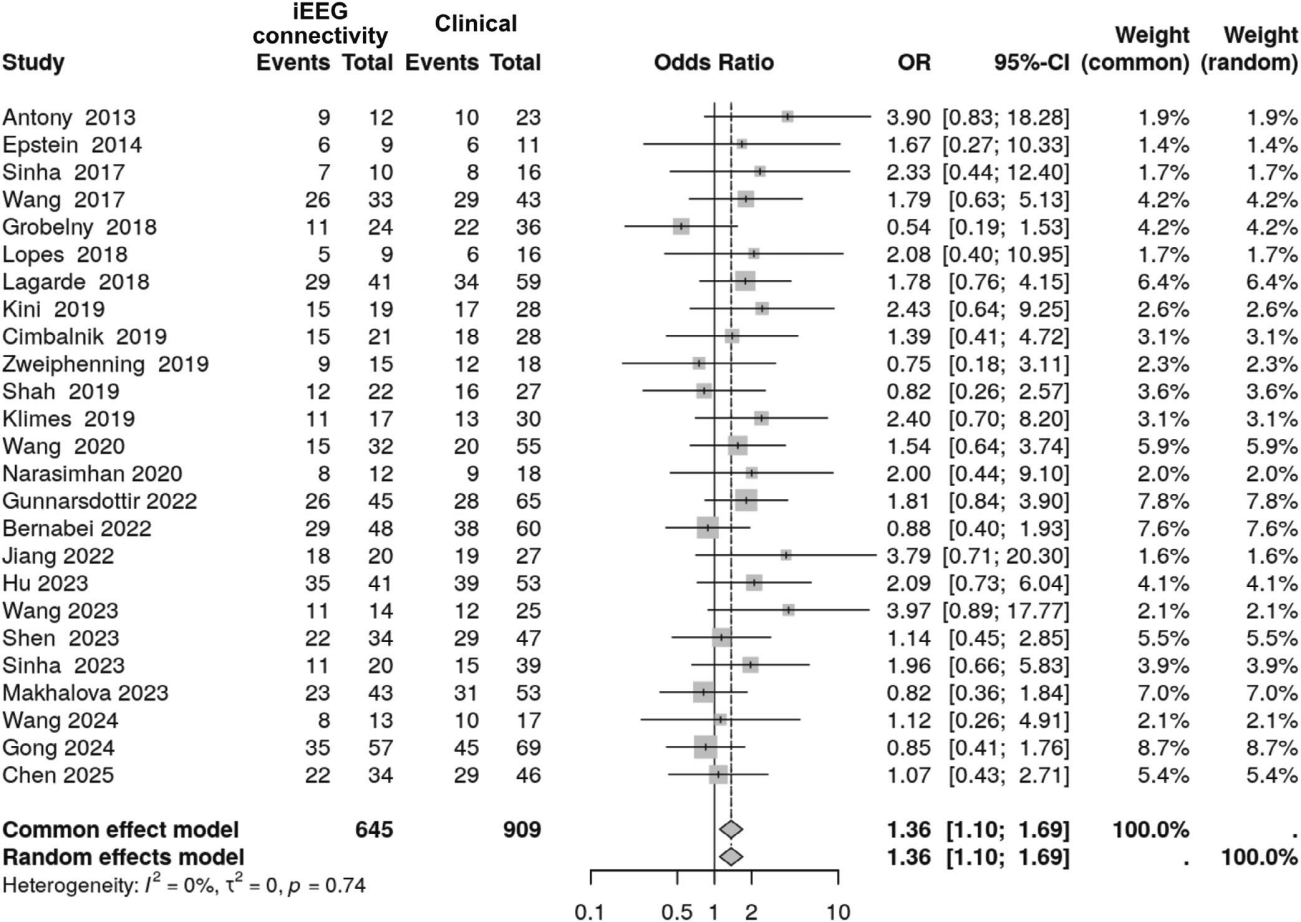
Forest plot of individual and pooled odds ratios comparing intracranial electroencephalographic (iEEG) connectivity to clinical evaluation across 25 studies. Each study's effect size is represented as an odds ratio (OR) with 95% confidence interval (CI) under a random effects model. The pooled estimate indicated a significant association in favor of iEEG connectivity (OR = 1.36, 95% CI = 1.10–1.69, *p* = .0048). Between‐study heterogeneity was negligible (*τ* ^2^ = 0, *I* ^2^ = .0%, *Q* = 19.19, *df* = 24, *p* = .742), suggesting consistency across studies. Meta‐analysis was conducted using the inverse variance method with restricted maximum‐likelihood estimation. Confidence intervals for *τ* ^2^ and *τ* were derived using the *Q*‐profile method.

A total of 25 studies were included in the meta‐analysis comparing the predictive performance of iEEG connectivity measures against clinical evaluation for localizing the epileptogenic zone. The pooled OR under a random effects model was 1.36 (95% CI = 1.10–1.69, *p* = .0048), indicating a significant advantage of additional iEEG connectivity measures. Between‐study heterogeneity was negligible (*τ* ^2^ = 0, *I* ^2^ = 0%, *Q* = 19.19, *df* = 24, *p* = .742), supporting consistency across studies (Figure 2).

### 3.2 Subgroup analyses

#### 3.2.1 Connectivity type: Directed versus undirected

**FIGURE 3:**
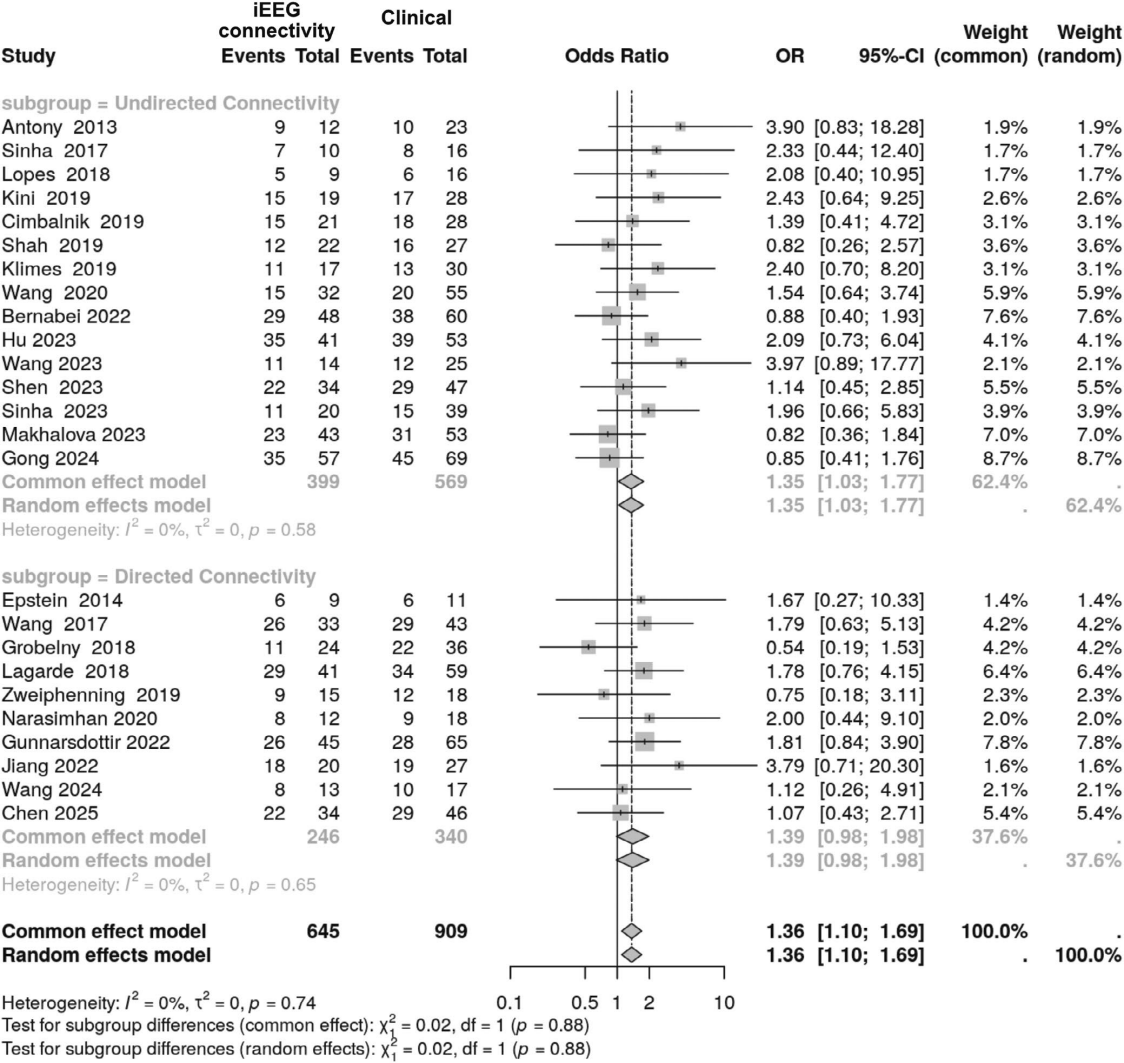
Subgroup analysis of odds ratios for intracranial electroencephalographic (iEEG) connectivity versus clinical evaluation, categorized by connectivity type directed versus undirected. The forest plot displays pooled and individual odds ratios (ORs) with 95% confidence intervals (CIs) for 25 studies, grouped by whether iEEG connectivity was measured using directed or undirected methods. The pooled effect size for studies using undirected connectivity was OR = 1.35 (95% CI = 1.03–1.77), whereas for directed connectivity the pooled OR was 1.39 (95% CI = .98–1.98), both estimated using a random effects model. Between‐study heterogeneity was negligible in both subgroups (*I* ^2^ = 0%, *τ* ^2^ = 0). The test for subgroup differences was not statistically significant (χ^2^ = .02, *df* = 1, *p* = .88), suggesting no meaningful difference in effect size between directed and undirected iEEG measures.

Subgroup analysis based on the type of connectivity revealed that undirected methods (*n* = 15 studies) yielded a pooled OR of 1.35 (95% CI = 1.03–1.77), whereas directed methods (*n* = 10 studies) showed an OR of 1.39 (95% CI = .98–1.98). There was no statistically significant subgroup difference (χ^2^ = .04, *df* = 1, *p* = .84; Figure 3).

#### 3.2.2 Epoch type: Interictal versus peri‐ictal

**FIGURE 4:**
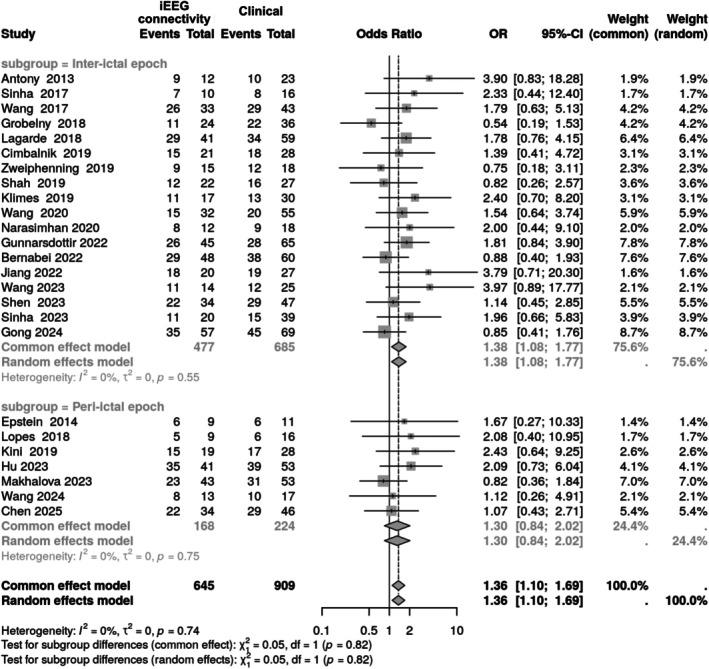
Subgroup analysis of odds ratios for intracranial electroencephalographic (iEEG) connectivity versus clinical evaluation, categorized by EEG segment type (interictal vs. peri‐ictal epochs). The forest plot displays effect sizes (odds ratios [ORs] with 95% confidence intervals [CIs]) for 25 studies grouped by whether iEEG connectivity was derived from interictal or peri‐ictal data. The pooled odds ratio for interictal studies was 1.38 (95% CI = 1.08–1.77), whereas for peri‐ictal studies it was 1.30 (95% CI = .84–2.02), both estimated using a random effects model. Heterogeneity was negligible within both subgroups (*I* ^2^ = 0%, *τ* ^2^ = 0). No statistically significant subgroup difference was observed (χ^2^ = .05, *df* = 1, *p* = .82), indicating similar predictive value regardless of iEEG segment timing.

When stratified by EEG epoch, studies using interictal segments (*n* = 18) showed a significant pooled OR of 1.38 (95% CI = 1.08–1.77), whereas peri‐ictal segments (*n* = 7) yielded a nonsignificant OR of 1.30 (95% CI = .84–2.02). However, no significant difference was observed between the two subgroups (χ^2^ = .03, *df* = 1, *p* = .85; Figure 4).

#### 3.2.3 Connectivity modeling: Linear versus nonlinear

**FIGURE 5:**
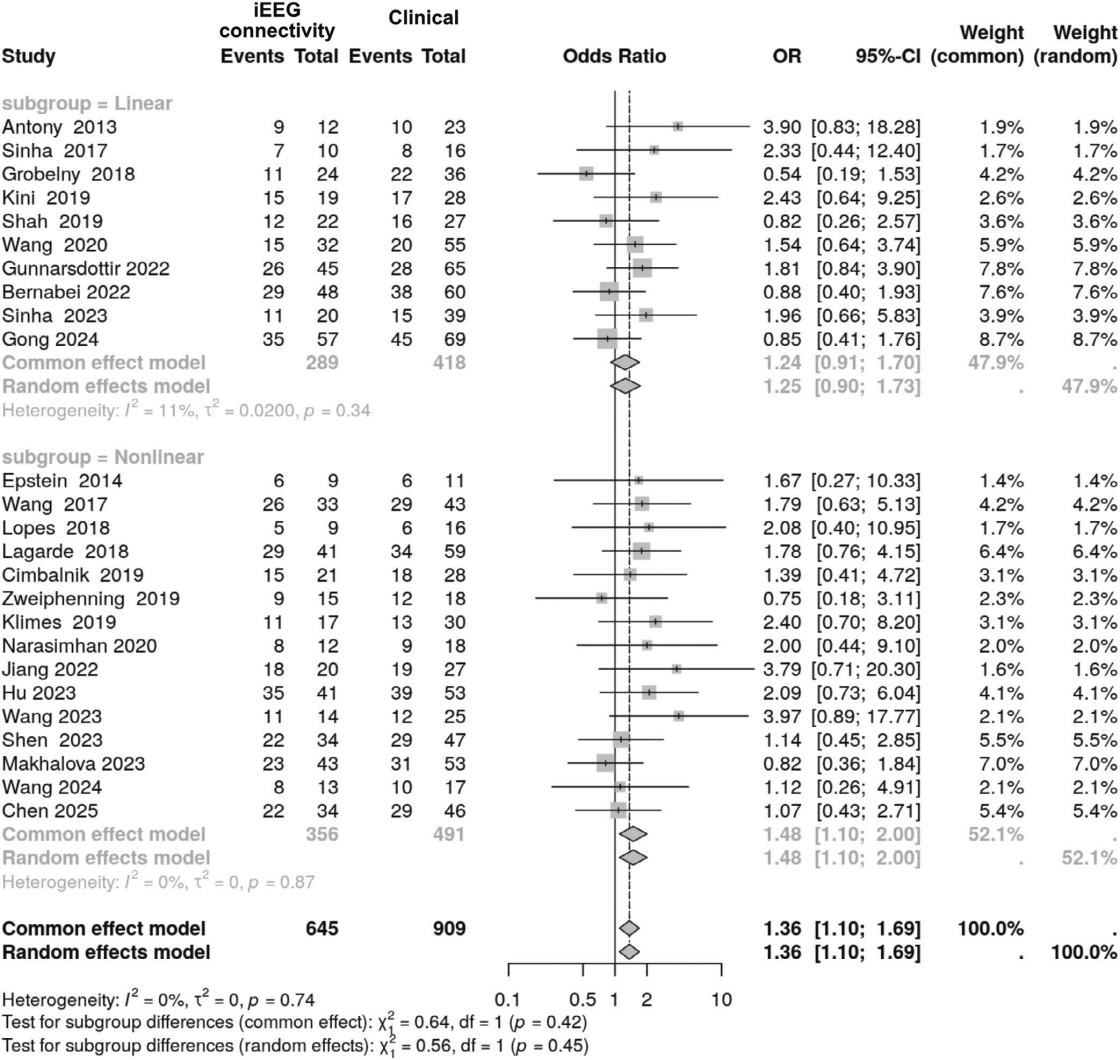
Subgroup analysis of odds ratios for intracranial electroencephalographic (iEEG) connectivity versus clinical evaluation, categorized by connectivity method (linear vs. nonlinear). This forest plot presents individual and pooled effect sizes for 25 studies, grouped by whether iEEG connectivity was estimated using linear or nonlinear methods. Studies using linear connectivity methods yielded a pooled odds ratio (OR) of 1.25 (95% confidence interval [CI] = .90–1.73), whereas nonlinear methods showed a higher pooled effect (OR = 1.48, 95% CI = 1.10–2.00), both under random effects models. Heterogeneity within subgroups was low (*I* ^2^ = 11% for linear, *I* ^2^ = 0% for nonlinear). However, subgroup difference tests were not statistically significant (χ^2^ = .56, *df* = 1, *p* = .45), suggesting no clear differential predictive value between linear and nonlinear modeling approaches.

Studies using nonlinear methods (*n* = 15) reported a higher pooled OR of 1.48 (95% CI = 1.10–2.00) compared to linear methods (*n* = 10), which produced a nonsignificant pooled OR of 1.24 (95% CI = .91–1.70). Nonetheless, the test for subgroup differences was not significant (*χ* ^2^ = .56, *df* = 1, *p* = .45; Figure 5).

### 3.3 Influence of patient characteristics on the observed benefit of iEEG connectivity

We investigated whether differences in patient populations could explain variation in how much benefit studies reported from using iEEG connectivity compared to clinical evaluation. Specifically, we examined whether the proportion of patients who were not seizure‐free after surgery—stratified by epilepsy type (temporal vs. extratemporal) and magnetic resonance imaging lesion status (lesional vs. nonlesional)—was associated with the observed effect size.

Across 11 studies with available data, we found that studies with a higher proportion of non‐seizure‐free patients with TLE reported a greater added value of iEEG connectivity over clinical evaluation alone (*β* = 3.53, 95% CI = .27–6.78, *p* = .038). A similar pattern was seen for lesional epilepsy, where studies with more non‐seizure‐free patients also showed larger effect sizes (*β* = 2.30, 95% CI = .19–4.40, *p* = .037). These findings suggest that iEEG connectivity may be mostly beneficial when standard clinical evaluation is less predictive of outcome.

In contrast, the proportions of non‐seizure‐free patients with extratemporal lobe epilepsy (*β* = −.63, *p* = .471) and nonlesional epilepsy (*β* = −1.74, *p* = .123) were not significantly associated with effect size. This indicates that the added value of iEEG connectivity in these subgroups was more variable and did not follow a consistent pattern. These results may be influenced by the small number of studies and overlapping clinical characteristics.

**FIGURE 6:**
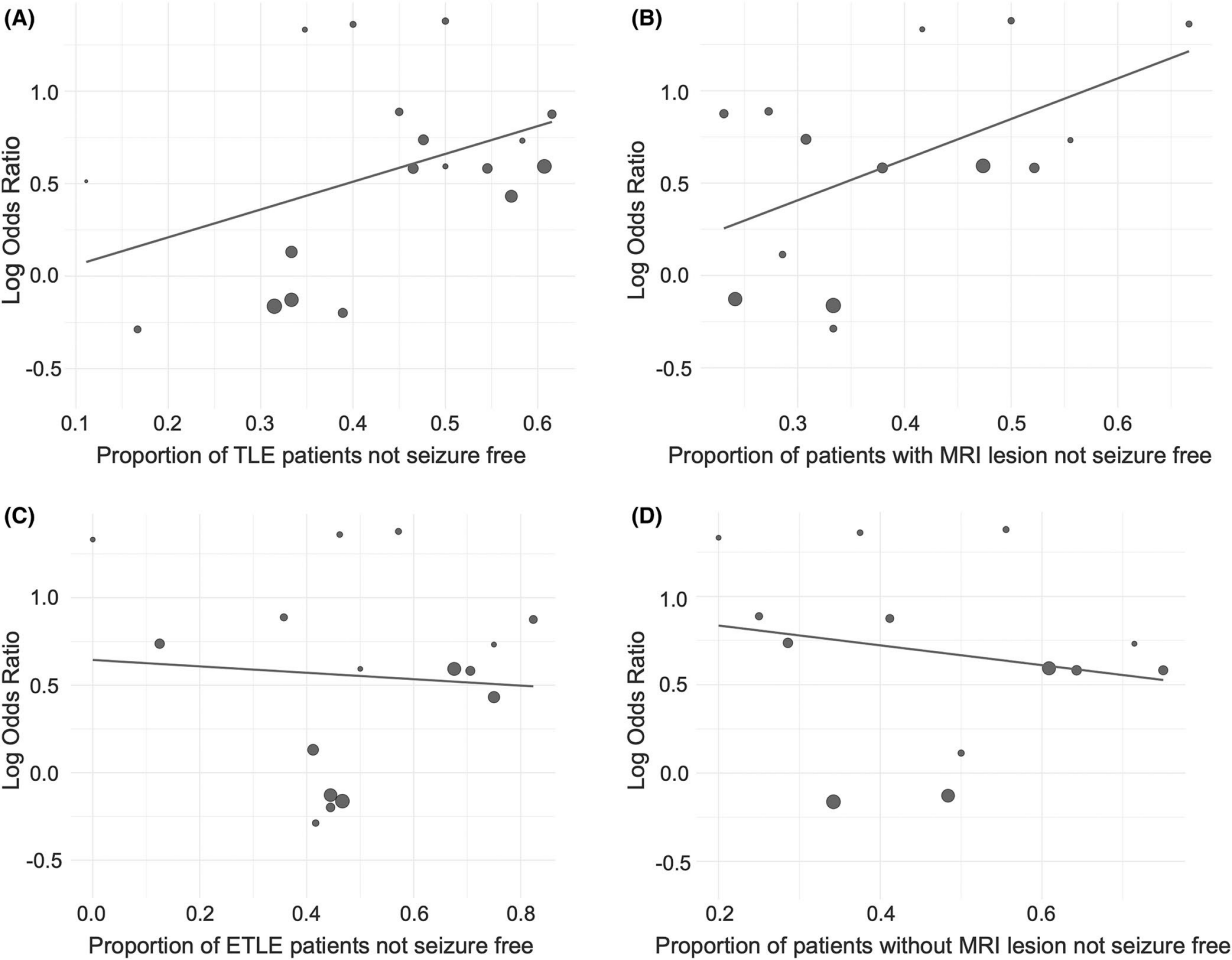
Bubble plots showing relationship between proportion of patients not seizure‐free (NSF) in each study and the observed log odds ratio comparing the added value of iEEG connectivity methods to clinical methods. Each point represents a study, with bubble size proportional to inverse‐variance weight. Results are shown for (A) temporal lobe epilepsy (TLE) NSF proportion, (B) lesional NSF proportion, (C) extratemporal lobe epilepsy (ETLE) NSF proportion, and (D) nonlesional NSF proportion. Proportions were calculated as NSF / (SF + NSF) within each subgroup, where SF = seizure‐free. In a robust meta‐regression model including all four variables, studies with a higher proportion of non‐seizure‐free patients with TLE (*β* = 3.53, 95% CI = .27–6.78, *p* = .0378) and lesional epilepsy (*β* = 2.30, 95% CI = .19–4.40, *p* = .0368) showed significantly greater benefit of iEEG connectivity analysis methods. ETLE (*p* = .471) and nonlesional (*p* = .123) NSF proportions were not significantly associated with effect size. This apparent specificity in TLE may reflect involvement of mesial temporal structures, which are often difficult to distinguish as seizure onset versus early spread regions. MRI, magnetic resonance imaging.

The meta‐regression model explained all between‐study differences in effect size (*τ* ^2^ = 0, *I* ^2^ = 0%, *R* ^2^ = 100%), indicating a strong overall model fit. The meta‐regression findings may be interpreted as exploratory, because there is no between‐study variance to explain. Although the model indicates an excellent model fit, these results should be interpreted with caution, because the limited number of studies (*n* = 11) and potential correlation among moderators may have artificially minimized between‐study variance. Full statistical results are provided in Supplementary Table 2 and visualized using bubble plots in Figure 6, where each point represents one study.

### 3.4 Assessment of publication bias

#### 3.4.1 Funnel plot asymmetry and Egger test

**FIGURE 7:**
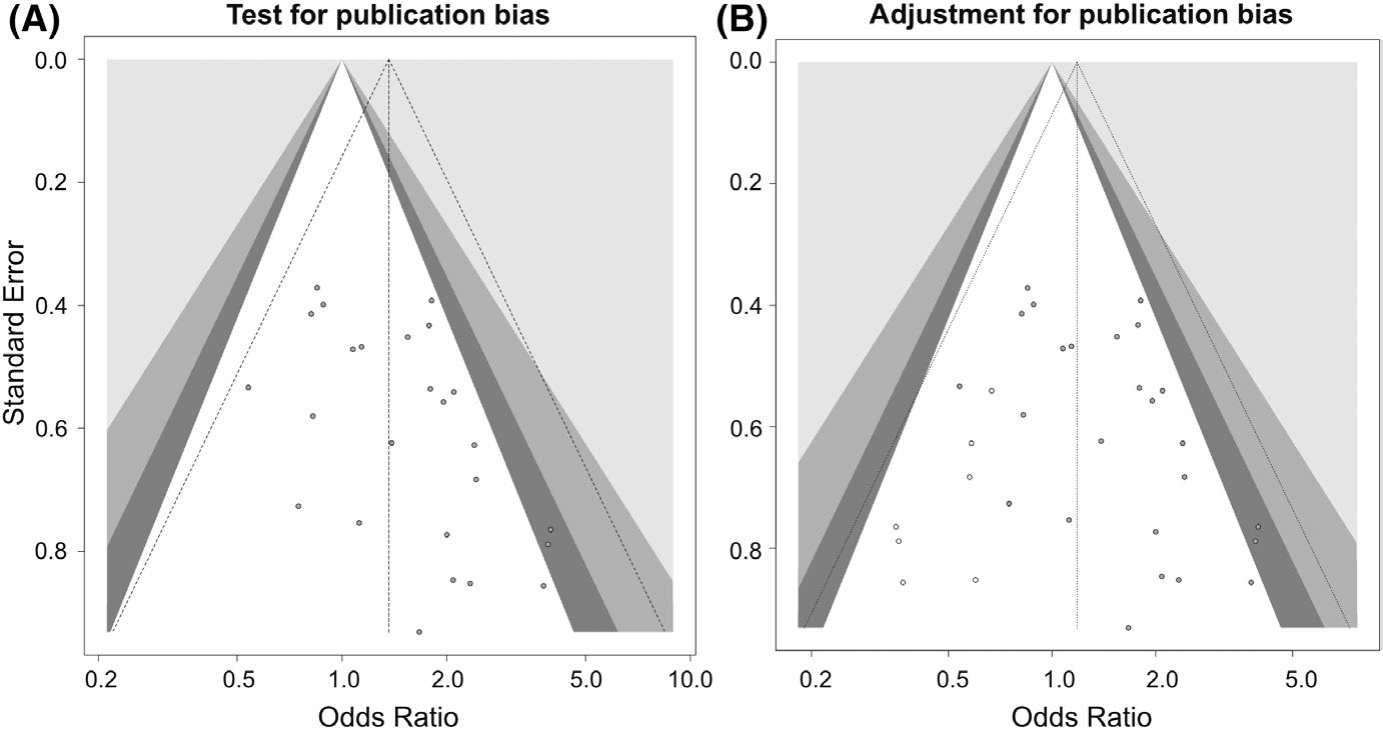
Funnel plots illustrating the detection and adjustment of publication bias using the Trim and Fill method. (A) Funnel plot assessing potential publication bias in the original dataset. Visual asymmetry is observed, and Egger regression test indicates significant asymmetry (*p* = .01), suggesting the presence of publication bias. (B) Funnel plot after applying the Trim and Fill method, which imputed seven potentially missing studies to restore symmetry. The adjusted meta‐analysis included 32 studies (25 original + 7 imputed), yielding an odds ratio of 1.18 (95% confidence interval = .97–1.44) under both common effect and random effects models (*p* = .10). Heterogeneity was low (*I* ^2^ = 2.1%); we applied inverse variance method with a restricted maximum likelihood estimator for between‐study variance.

Visual inspection of the funnel plot (Figure 7A) suggested asymmetry, indicating potential publication bias. This was supported by Egger regression test (*p* = .01), indicating that smaller studies with null findings may be underrepresented.

#### 3.4.2 Trim and fill adjustment

To correct for funnel plot asymmetry, the Trim and Fill method was applied, imputing seven potentially missing studies, resulting in an adjusted meta‐analysis of 32 studies. The adjusted pooled OR remained nonsignificant at 1.18 (95% CI = .97–1.44, *p* = .10) under a random effects model, with low heterogeneity (*I* ^2^ = 2.1%; Figure 7B). This adjustment suggests that although publication bias may exist, it does not fully account for the observed effect size.

## 4 DISCUSSION

Over the past 2 decades, numerous studies have proposed iEEG connectivity methods as tools to quantitatively localize epileptic networks for surgical planning. In this systematic review and meta‐analysis, we found that iEEG connectivity approaches were associated with a 36% improvement in seizure outcome prediction compared to standard clinical evaluation to guide surgical resection or ablation to control seizures (pooled OR = 1.36, 95% CI = 1.10–1.69). This supports a consistent overall benefit of incorporating connectivity analyses into presurgical assessment. However, at the individual study level, most estimates were not statistically significant, with CIs for ORs commonly crossing 1.0. This finding reflects limited sample sizes in nearly all published studies, which constrained statistical power and masked subgroup effects. The lack of individual participant‐level data further impedes our ability to determine which types of patients may benefit most from these methods. Although our analysis demonstrates a statistically significant effect at the group level, future progress will require systematic improvements in study design, including larger sample sizes, standardized reporting, and multicenter data sharing to support clinical translation.

### 4.1 Sample size requirements and power considerations

**FIGURE 8:**
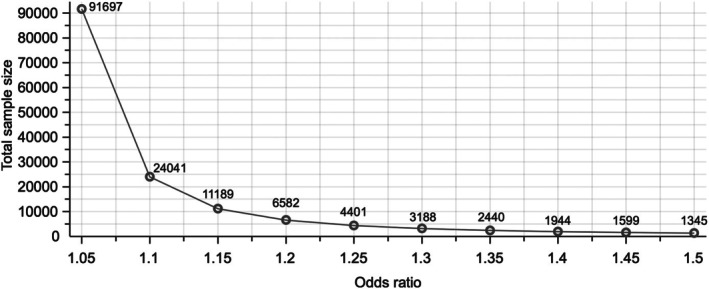
Sample size needed to detect significant effect. Power analysis was conducted using G*Power (test family: Exact, test type: Proportions—inequality, two independent groups, two‐tailed). Parameters included *α* = .05, power = .95, group 2 proportion = .5, and an allocation ratio (N2/N1) of .65. The figure illustrates the total sample size required to detect a range of odds ratios. As effect sizes decrease (i.e., odds ratios approach 1.0), the required sample size increases sharply, with more than 90 000 participants needed to detect an odds ratio of 1.05, and approximately 1345 for an odds ratio of 1.5. These sample size estimates far exceed those of any individual study in our meta‐analysis, underscoring the importance of collaborative data sharing and the need for multicenter efforts to achieve adequate power.

A major limitation across the published literature related to iEEG is low statistical power due to small sample sizes. Even studies with promising effects had small sample sizes, limiting the ability to detect subgroup effects or generalizable findings. Guided by this meta‐analysis, we conducted a power analysis to determine the sample size needed to detect a clinically significant effect (Figure 8). Assuming *α* = .05 and 95% power, to achieve a 50% improvement in surgical outcomes from iEEG connectivity methods (OR = 1.5) would require approximately 1300 participants, far exceeding the size of any individual study in our review. These findings highlight the necessity of multicenter collaborations and shared data repositories to achieve adequate power. Without such infrastructure, the field risks stagnation despite encouraging preliminary evidence.^42^

### 4.2 Recommendations to improve study design

Our analysis underscores several opportunities to improve study design and reporting to evaluate iEEG connectivity methods. First, there is a critical need for greater transparency and consistency in reporting outcomes. Among the 25 included studies, only four provided individual participant data (IPD), totaling 51 patients (Supplementary Text). This limited dataset constrained our ability to detect statistically significant associations between clinical characteristics and seizure outcomes in patients where iEEG connectivity findings were concordant with clinical localization.

To advance the field, we recommend that future studies align with PRISMA‐IPD and ILAE guidelines by systematically reporting patient‐level concordance or discordance between iEEG‐derived targets and clinical localization. This should include the following: (1) whether the iEEG connectivity‐defined region was surgically resected in individual patients; (2) whether postoperative seizure freedom was achieved on Engel or ILAE scales in individual patients; (3) key clinical metadata (e.g., lesion status, localization type, modality [SEEG vs. ECoG]), along with other demographic variables; and (4) confusion matrix (TP, FP, FN, TN), thresholds and scores at the individual patient level.^43^, ^44^

Standardized and complete reporting of such data is essential to enable future pooled IPD meta‐analyses and to identify subgroups, such as patients with nonlesional epilepsy or extratemporal localization, who may benefit most from iEEG connectivity‐guided approaches. Ultimately, harmonizing data collection and sharing across centers will be essential for progress toward clinical trials and evidence‐based adoption.

An additional methodological consideration concerns the exclusion of TN cases from performance assessment. In retrospective studies, such cases are inherently indeterminate, because it is impossible to know whether removing regions suggested by iEEG connectivity—rather than those selected clinically—would have led to seizure freedom. Including them would require counterfactual assumptions and introduce bias. Only prospective studies, in which surgical targets are defined a priori based on connectivity findings and outcomes are systematically tracked, can validly assess TN and estimate specificity.

Palliative or functionally constrained surgeries are an important consideration when interpreting connectivity concordance. Such procedures, often limited for safety or functional reasons, may yield poor outcomes despite accurate localization. In this meta‐analysis, clinical localization was defined as the surgically treated region (resection or ablation). We recommend that future research explicitly distinguish curative from palliative surgeries to improve interpretation of concordance and guide clinical translation.

### 4.3 Methodological limitations

Our findings must be interpreted in light of several methodological limitations. First, although we identified a significant group‐level effect, individual studies were generally underpowered, with most failing to demonstrate statistical significance on their own. This was compounded by variability in definitions of good seizure outcomes, inconsistent use of Engel and ILAE scales, heterogeneity across connectivity methods, and lack of standardized thresholds for evaluating iEEG connectivity‐derived targets.

Additionally, the retrospective nature of these studies limits interpretation of TN cases, as it is impossible to know whether alternative regions suggested by iEEG connectivity would have yielded better outcomes if resected or ablated. It is also possible that small sample sizes, residual confounds, and incomplete clinical variables may also have limited the generalizability of these findings. Although we applied mixed‐effects logistic regression to account for clustering by study, the lack of measurable between‐study variability suggests that heterogeneity may not be adequately modeled, likely a result of underpowered subgroup comparisons. Finally, publication bias remains a concern, as indicated by asymmetry in the funnel plot and Egger test, although adjusted estimates remained broadly consistent with the primary analysis.

Variation in postoperative follow‐up duration across studies may also influence outcome estimates, as early seizure freedom does not necessarily predict long‐term surgical success; detailed follow‐up information was inconsistently reported and therefore could not be analyzed quantitatively in this meta‐analysis.

It is also important to note that this analysis does not position iEEG connectivity as an independent comparator to standard clinical evaluation. Rather, connectivity findings are inherently influenced by clinical decision‐making, particularly in the selection of electrode implantation sites, which is guided by presurgical hypotheses. As such, our findings reflect the added value of performing iEEG connectivity analysis as a complement to clinical evaluation, not as a replacement. We do not claim that iEEG connectivity methods are superior to standard clinical approaches but instead highlight their potential to augment surgical planning in select contexts.

This meta‐analysis was restricted to adult patients, as pediatric drug‐resistant epilepsy differs fundamentally in neurodevelopment, etiology, and clinical management. Ongoing brain maturation, distinct pathological substrates, and differing surgical strategies can alter both iEEG signal characteristics and the interpretation of connectivity measures. Including pediatric data would have introduced substantial clinical and statistical heterogeneity. Future work should address pediatric cohorts separately using age‐appropriate criteria and outcome metrics.

### 4.4 Impact on future research direction

This meta‐analysis highlights several actionable priorities for future research to accelerate the clinical translation of iEEG connectivity methods. First, undersampled studies must be interpreted with caution. Although many included studies report promising trends, nearly all were statistically underpowered at the individual level. Our analysis confirms that no single study demonstrated a significant benefit of iEEG connectivity methods on its own. The added value of these approaches only became evident when data were pooled across studies. This underscores a critical lesson; in translational neuroscience, no single “high‐impact” study is sufficient. Rather, robust evidence emerges from synthesizing signals across multiple rigorously conducted investigations.

To enable this synthesis, public sharing of raw data is essential.^42^, ^45^, ^46^ Funders, institutions, and journals should require open‐access publication of minimally deidentified, patient‐level data. Such transparency is particularly vital for complex analytic methods, like iEEG connectivity, where outcome interpretation depends heavily on methodological details, validated comparative analysis, and concordance with surgical decisions. The continued accumulation of small, siloed datasets without public access significantly limits the field's ability to identify reliable biomarkers and translate them into clinical tools.

Third, clinical research should prioritize the inclusion of underrepresented subgroups. Our meta‐regression showed that studies with higher proportions of non‐seizure‐free patients with TLE and lesional epilepsy demonstrated greater benefit from iEEG connectivity. However, data were too sparse to evaluate the added value of these methods in patients with extratemporal or nonlesional epilepsy, precisely the populations where standard evaluation is least reliable. This represents a missed opportunity. Future studies must intentionally recruit and report on these patient subgroups and ensure that individual participant data, including concordance with clinical localization and outcome classification, are made available for pooled analyses.

Fourth, editors and reviewers must uphold rigorous standards for reporting and transparency. Studies should not be published based solely on novelty or group‐level accuracy metrics but must demonstrate methodological reproducibility and include adequate patient‐level metadata that align with FAIR (Findable, Accessible, Interoperable, and Reproducible) data practices.^47^ Without these safeguards, the field risks generating isolated findings that cannot be validated or implemented at scale.

Finally, publication bias remains a concern, as studies with negative or nonconfirmatory results are less likely to be reported. We strongly recommend that all studies, regardless of outcome, be published and shared. Balanced reporting—supported by authors, editors, and reviewers—is essential to reduce bias and build a more reliable, evidence‐based understanding of iEEG connectivity in epilepsy surgery.

### 4.5 Conclusions

This meta‐analysis synthesizes 2 decades of research on iEEG connectivity and provides the first quantitative estimate of its added value over standard clinical evaluation alone for epilepsy surgery planning. Although pooled results demonstrate a statistically significant 36% improvement in seizure outcome prediction, individual studies remain underpowered, and key patient subgroups—particularly those with extratemporal or nonlesional epilepsy—are insufficiently represented. Our findings underscore that the clinical utility of iEEG connectivity methods cannot be assessed from isolated studies alone; rather, meaningful progress requires multicenter collaboration, standardized data reporting, and public sharing of individual‐level data. To ensure reproducibility and advance toward clinical translation, future studies must adhere to rigorous methodological frameworks, expand inclusion of complex cases, and contribute openly to shared datasets that can be integrated with other multimodal epilepsy data. These recommendations are essential not only for validating iEEG connectivity as a clinical tool but also for shaping a more robust, inclusive, and data‐driven future for translational epilepsy research.

## AUTHOR CONTRIBUTIONS

Nishant Sinha contributed to conceptualization, methodology, study design, data curation, formal analysis, visualization, interpretation of data, writing of the original draft, writing–review and editing, and supervision. Daniel J. Zhou contributed to conceptualization, methodology, study design, data curation, formal analysis, visualization, and writing–review and editing. Victoria L. Morgan contributed to conceptualization, methodology, interpretation of data, and writing–review and editing. Dario J. Englot, Leonardo Bonilha, Milan Brázdil, Xiaosong He, Riki Masumoto, Terence J. O'Brien, Chengyuan Wu, Erik Kaestner, Carrie McDonald, Ezequiel Gleichgerrcht, and Aileen McGonigal contributed to conceptualization, methodology, and writing–review and editing. Quy Cao contributed to conceptualization, methodology, formal analysis, and writing–review and editing. Kathryn A. Davis contributed to conceptualization, methodology, interpretation of data, writing–review and editing, and supervision.

## FUNDING INFORMATION

N.S. received funding from the National Institute of Neurological Disorders and Stroke (NINDS) of the National Institutes of Health under award numbers K99NS138680 (principal investigator [PI]: Sinha), R01NS125137 (PI: Litt), and Department of Defense W81XWH2210593 (PI: Sinha). K.A.D. received funding from NINDS R01NS116504 (PI: Davis), R61NS125568 (PI: Davis), and U24NS134536 (PI: Wagenaar). X.H. received funding from the National Natural Science Foundation of China (grant Nos. 82271491 and W2521183). T.J.O. received funding from the NHMRC Investigator Grant Scheme (APP1176426 and APP2034258).

## CONFLICT OF INTEREST STATEMENT

None of the authors has any conflict of interest to disclose. We confirm that we have read the Journal's position on issues involved in ethical publication and affirm that this report is consistent with those guidelines.

## Supporting information


Data S1.

Table S1.

## Data Availability

Data extracted and analyzed for this systematic review and meta‐analysis are fully reported within the article, figures, and supplementary tables. No new primary data were generated for this study.
