# Supplementary material for: Intracranial electroencephalographic connectivity analysis to localize epileptogenic networks: Systematic review and meta‐analysis from ILAE Epilepsy Surgery Networks Task Force
Source: Epilepsia. 2026 Mar 2;67(6):2707–24. doi: 10.1002/epi.70168 (PMC13285243; doi:10.1002/epi.70168)
Supplement: Supplementary file 1 — Data S1. [file EPI-67-2707-s002.pdf]

Supplementary Materials

Supplementary: Search Strategy

PubMed

((((("connectome"[MeSH Terms] OR "connectome"[tw] OR "connectomes"[tw] OR "connectomic"[tw] OR "connectomics"[tw]) OR ("brain mapping"[MeSH Terms] OR ("brain"[tiab] AND "mapping"[tiab]) OR "brain mapping"[tiab])) OR (networks [tw] OR network [tw] OR nerve net [tiab] OR connectivity [tiab])) AND ((("Epilepsy"[Mesh:NoExp]) OR "Drug Resistant Epilepsy"[Mesh]) OR "Epilepsies, Partial"[Mesh] OR epilepsy [tiab] OR seizure [tiab] OR seizures [tiab])) AND ("surgery"[MeSH Subheading] OR "surgical procedures, operative"[MeSH Terms] OR "general surgery"[MeSH Terms] OR "surgery"[tiab] OR "surgical"[tiab] OR "surgery s"[tiab] OR "surgerys"[tiab] OR "surgeries"[All Fields])) AND ((("Electrocorticography"[Mesh] OR Electrocorticographies [tiab] OR Electrocorticography [tiab] OR "intracranial EcoG" [tiab] OR "intracranial EcoGs" [tiab] OR "intracranial EEG" [tiab] OR "intracranial EEGs" [tiab])) OR ((Intracranial Electroencephalography) [tiab] OR "Electrocorticography"[Mesh] OR (Intracranial Electroencephalographies) [tiab] OR Stereoelectroencephalography OR SEEG [tiab]))

Embase

Step	Search Syntax
1	'connectome'/exp OR 'connectivity map (neural circuit)' OR 'connectomic' OR 'connectomics' OR 'connectome' OR connectomes
2	'brain mapping'/exp OR 'brain mapping':ti,ab OR (brain:ti,ab AND mapping:ti,ab)
3	networks:ti,ab OR network:ti,ab OR (nerve:ti,ab AND net:ti,ab) OR connectivity:ti,ab
4	#1 OR #2 OR #3
5	'epilepsy'/de OR 'refractory epilepsy'/exp OR 'focal epilepsy'/exp OR epilepsy:ti,ab OR seizure:ti,ab OR seizures:ti,ab

6	'surgery'/exp OR 'general surgery'/exp OR surgical:ti,ab OR surgery?s:ti,ab OR surgery:ti,an OR surgeries:ti,ab
7	'electrocorticography'/exp OR electrocorticography:ti,an OR electrocorticographies:ti,ab OR 'intracranial ecog':ti,ab OR 'intracranial ecogs':ti,ab OR 'intracranial eeg':ti,ab OR 'intracranial eegs':ti,an OR 'intracranial electroencephalography':ti,ab OR 'intracranial electroencephalographies':ti,ab OR stereoelectroencephalography:ti,ab OR seeg:ti,ab
8	#4 AND #5 AND #6 AND #7

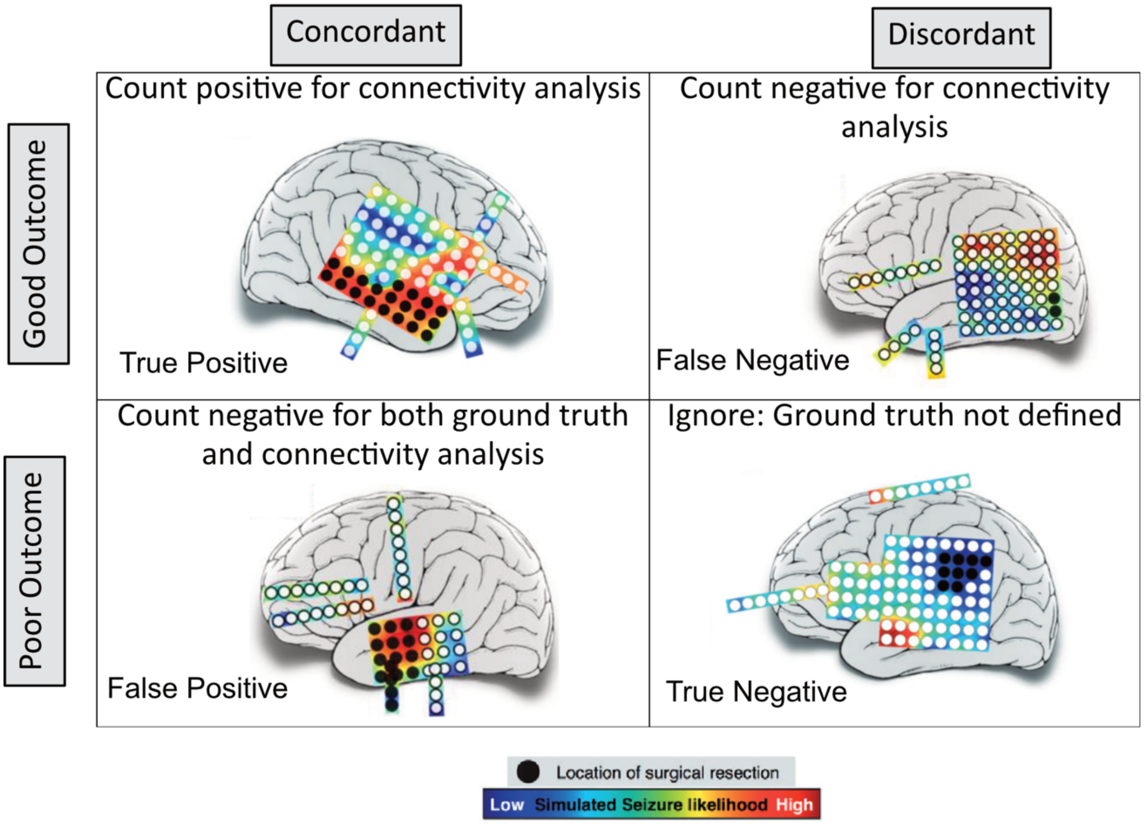

Supplementary Figure 1. Example classification of patient outcomes based on concordance between iEEG connectivity-derived targets and clinical resection zones, overlaid on cortical maps. This figure illustrates four example cases corresponding to the true positive (TP), false negative (FN), false positive (FP), and true negative (TN) categories used to evaluate the added value of iEEG connectivity analysis. Each panel shows the brain of a patient implanted with electrocorticography (ECoG) electrodes from an illustrative study (Sinha 2017)[23], annotated with the simulated seizure likelihood based on iEEG connectivity analysis (color-coded from blue to red), and the region of surgical resection (outlined in black). Top Left (TP): In this case, connectivity-derived targets concord with the resected region, and the patient achieved a good outcome. These cases are counted as true positives and considered supportive of the clinical value of iEEG connectivity analysis. Top Right (FN): Connectivity analysis fails to identify the clinically targeted region, and the patient had a poor outcome. This is classified as a false negative, indicating missed localization by the connectivity method. Bottom Left (FP): The connectivity analysis identifies a region that overlaps with the clinically resected zone, but the patient had a poor outcome. Although both connectivity and clinical localization agree, the lack of benefit suggests the region was not epileptogenic. This is labeled a false positive in the connectivity context. Bottom Right (TN): Connectivity and clinical targets do not concord, and the outcome was also poor. These true negative cases are excluded from the main analysis because, in a retrospective framework, it is not possible to determine whether resecting the connectivity-identified region would have led to a better outcome. The heatmaps illustrate one way in which iEEG connectivity methods attempt to quantify seizure likelihood by measuring signal interactions

across brain regions. However, connectivity-derived targets are not uniformly defined across studies. Different methods— like cross-correlation, Granger causality, entropy measures, among others—were used to infer network properties, leading to variability in what is considered the "epileptogenic network" across studies. Our meta-analysis found variation in performance across methods, but no single approach showed statistically significant clinical benefit on its own, as most effect estimates crossed an odds ratio of 1. This highlights that while methodological choices may influence performance, improved algorithms alone—especially in small datasets—are unlikely to ensure generalizability. Moreover, none of the included studies used iEEG connectivity prospectively to guide surgery, except (Epstein 2014)[24] in which two cases were prospectively tested. Future prospective studies will be necessary to adjudicate indeterminate categories like true negatives, and to validate whether targeting iEEG connectivity-identified regions can improve outcomes. Note that while this figure uses ECoG recordings for visualization, our meta-analysis included both ECoG and SEEG studies. Where individual participant data were available, we examined whether implantation scheme moderated the predictive value of connectivity analysis.

Supplementary Glossary

- *iEEG connectivity-derived targets:* Brain regions identified as functionally or causally connected within the epileptogenic network, derived from iEEG-based graph models in which nodes represent recording sites and edges represent measures of connectivity (e.g., correlation, coherence, directed transfer function, Granger causality).
- *Concordance with clinical evaluation (operational criteria):* Concordance and discordance were defined within the framework of a confusion matrix, linking connectivity-derived targets, clinical localization, and surgical outcomes. Each patient case was categorized as one of four possible outcomes: True Positive (TP) – connectivity and clinical localization concordant, good outcome; False Positive (FP) – connectivity and clinical localization concordant, poor outcome; False Negative (FN) – connectivity and clinical localization discordant, good outcome; True Negative (TN) – connectivity and clinical localization discordant, poor outcome. The TP, FP, and FN categories were used to assess the added value of connectivity analysis, while TN cases were considered indeterminate due to the absence of ground truth in retrospective data.
- *Events:* Refers to the total number of patient-level observations included in each analysis, representing the count of cases contributing to each classification category (true positives, false positives, or false negatives). “Total events” corresponds to the sum of all such cases within a study or meta-analytic comparison. In this context, “events” denote patient outcomes contributing to statistical models, not electrophysiological signal events recorded during iEEG.

Supplementary: Individual Participant-Level Analysis

A mixed-effects logistic regression model was used to explore whether clinical characteristics predicted seizure freedom in cases where IEEG connectivity findings were concordant with clinical localization (i.e., true positive cases). Predictors included lesion status (lesional vs. non-lesional), surgery location (temporal vs. other), and electrode type (SEEG vs. ECoG), with study ID included as a random intercept to account for clustering by study. The model was implemented using the pymer4 Python package, which provides a seamless interface to R's lme4::glmer() function, allowing generalized linear mixed models (GLMMs) to be run from within Python.

The analysis included 51 participants from 4 studies. None of the predictors reached statistical significance. The odds of IEEG concordance with seizure freedom were lower in lesional cases (OR = 0.56, 95% CI: 0.13 to 2.41, p = 0.434), and slightly higher in patients evaluated with SEEG (OR = 1.79, 95% CI: 0.42 to 7.76, p = 0.434), though both estimates had wide confidence intervals. The model's ability to discriminate between IEEG-concordant seizure-free outcomes and other cases was limited, with an area under the receiver operating characteristic (ROC) curve (AUC) of 0.585.

These findings suggest that, within this small and heterogeneous sample, routinely reported clinical variables alone may not be sufficient to predict when IEEG connectivity methods meaningfully improve presurgical decision-making. The analysis was constrained by the modest sample size and inconsistent availability of patient-level variables across studies. Nonetheless, these exploratory results highlight the importance of future prospective research with standardized, harmonized individual-level data to better identify which patients are most likely to benefit from IEEG-guided interventions.

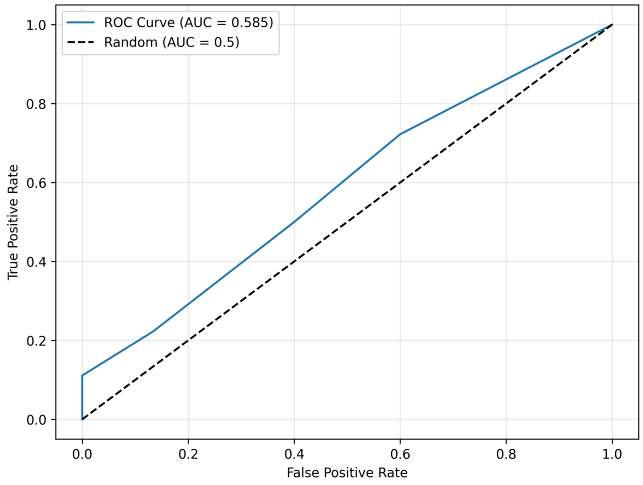

Supplementary Figure 2: ROC curve to determine added value of IEEG connectivity analysis for seizure freedom in individual participant with different clinical charecteristics: The ROC curve illustrates the classification performance of a logistic mixed-effects model predicting seizure freedom in patients where IEEG connectivity findings agreed with clinical localization (true positives). Predictors included lesion status (lesional vs. non-lesional), surgery location (temporal vs. other), and electrode type (SEEG vs. ECoG), with study ID as a random intercept. The area under the curve (AUC) was 0.585, indicating poor discriminative ability. This suggests that the clinical variables included in the model are not sufficient to reliably distinguish cases where IEEG connectivity adds value over standard clinical evaluation.

Supplementary Table 1: Fixed Effects Estimates from the Logistic Mixed-Effects Model

**Predictor**	**Estimate**	**OR**	**95% CI (OR)**	**p-value**
**Intercept**	1.31	3.70	[0.74, 18.58]	0.112
**Lesional (vs. non-lesional)**	-0.59	0.56	[0.13, 2.41]	0.434
**Temporal (vs. other)**	-0.22	0.81	[0.22, 2.94]	0.744
**SEEG (vs. ECoG)**	0.59	1.79	[0.42, 7.76]	0.434

Model details: binomial family with logit link; 51 patients from 4 studies; AIC = 70.46; random effect variance for study ID = 0.
