# Supplementary material for: Intracranial electroencephalographic connectivity analysis to localize epileptogenic networks: Systematic review and meta‐analysis from ILAE Epilepsy Surgery Networks Task Force
Source: Epilepsia. 2026 Mar 2;67(6):2707–24. doi: 10.1002/epi.70168 (PMC13285243; doi:10.1002/epi.70168)
Supplement: Supplementary file 2 — Table S1. [file EPI-67-2707-s001.pdf]

Supplementary Table 1. Joanna Briggs Institute (JBI) Critical Appraisal Checklist for Included Studies
This table summarizes the methodological quality assessment of all included studies using the Joanna Briggs Institute (JBI) Critical Appraisal Checklist for Case-Control Studies (Moola et al., 2020, JBI Manual for Evidence Synthesis) https://jbi.global/critical-appraisal-tools. Each study was appraised across ten criteria assessing comparability of groups, matching procedures, validity of exposure and outcome measurement, control of confounding, adequacy of exposure period, and appropriateness of statistical analyses.

Study (Year)	A	B	C	D	E	F	G	H	I	J	Total Score	Conclusion
Antony 2013	1	1	1	1	1	1	0	0	1	1	8	Include
Epstein 2014	1	0	0	1	1	0	0	1	1	1	6	Include
Sinha 2017	1	1	1	1	1	1	1	1	1	1	10	Include
Wang 2017	1	1	1	1	1	1	1	1	1	1	10	Include
Grobelny 2018	1	1	1	1	1	1	1	1	1	1	10	Include
Lopes 2018	1	1	1	1	1	0	1	1	1	1	9	Include
Lagarde 2018	1	1	1	1	1	1	1	1	1	1	10	Include
Kini 2019	1	1	1	1	1	1	0	1	1	1	9	Include
Cimbalnik 2019	1	0	1	1	1	0	0	1	1	1	7	Include
Zweiphenning 2019	1	1	1	1	1	1	0	1	1	1	9	Include
Shah 2019	1	0	1	1	1	0	0	1	1	1	7	Include
Klimes 2019	1	1	1	1	1	1	1	1	1	1	10	Include
Wang 2020	1	0	1	1	1	0	0	1	1	1	7	Include
Narasimhan 2020	1	0	1	1	1	0	0	1	1	1	7	Include
Gunnarsdottir 2022	1	1	1	1	1	1	1	1	1	1	10	Include
Bernabei 2022	1	1	1	1	1	1	1	1	1	1	10	Include
Jiang 2022	1	1	1	1	1	0	0	1	1	1	8	Include
Hu 2023	1	1	1	1	1	1	0	1	1	1	9	Include
Wang 2023	1	1	1	1	1	1	0	0	1	1	8	Include
Shen 2023	1	0	1	1	1	0	0	0	1	1	6	Include
Sinha 2023	1	1	1	1	1	1	1	1	1	1	10	Include
Makhalova 2023	1	1	1	1	1	1	1	1	1	1	10	Include
Wang 2024	1	0	1	1	1	0	0	1	1	1	7	Include
Gong 2024	1	1	1	1	1	1	1	1	1	1	10	Include
Chen 2025	1	0	1	1	1	0	0	1	1	1	7	Include

Checklist Items (1–10):

A. Groups comparable other than presence/absence of disease.
B. Cases and controls matched appropriately.
C. Same criteria used for identification of cases and controls.
D. Exposure measured in a valid and reliable way.
E. Exposure measured consistently for cases and controls.
F. Confounding factors identified.
G. Strategies to address confounding stated.
H. Outcomes assessed in a valid and reliable way.
I. Exposure period long enough to be meaningful.
J. Appropriate statistical analysis used.

Scoring: “1” = Yes, “0” = No/Unclear. Total score range: 0–10 points.
Quality interpretation: High (8–10), Moderate (5–7), Low (≤4).
All included studies demonstrated moderate-to-high methodological quality.
